# Lithium‐Ion Battery Safety Evaluation by Quantifying Lithium Plating Degree Based on Pressure Signals

**DOI:** 10.1002/advs.77129

**Published:** 2026-08-11

**Authors:** Haonan Liu, Jiangong Zhu, Wenyuan Weng, Bin Shen, Wenjun Fan, Yang Wang, Huapeng Lu, Wuliyasu He, Chunjing Lin, Xuezhe Wei, Haifeng Dai

**Affiliations:** ^1^ College of Automotive and Energy Engineering Tongji University Shanghai China; ^2^ Clean Energy Automotive Engineering Center Tongji University Shanghai China; ^3^ Volvo Car Technology (Shanghai) Co., Ltd Shanghai China; ^4^ Key Laboratory of Advanced Manufacturing Technology for Automobile Parts Ministry of Education Chongqing University of Technology Chongqing China

**Keywords:** Li‐plating degree, lithium‐ion battery, N/P ratio, pressure, thermal safety

## Abstract

Lithium (Li) plating poses a major challenge to the thermal safety of lithium‐ion batteries (LIBs). In this work, low N/P LIBs are constructed to induce controllable Li‐plating, and thermal response tests are performed to correlate Li‐plating degree with thermal safety characteristics. A quantitative descriptor of Li‐plating degree (*D*_Li_) is established, and post‐mortem characterizations show that increasing *D*_Li_ is accompanied by the accumulation of Li‐plating and side‐reaction products, along with aggravated interfacial degradation. Based on the evolution of pressure signals during calibration, a non‐destructive diagnostic model for *D*_Li_ is further developed. Accelerating rate calorimeter (ARC) results show that the first detectable self‐heating temperature of low N/P cells decreases monotonically with increasing *D*_Li_, indicating a progressively reduced thermal safety margin. Accordingly, *D*_Li_ thresholds of 20% and 30% are identified, and a thermal safety grading and battery management framework is established. Cross‐scale validation in commercial 12 Ah cells shows that the predicted *D*_Li_ values and safety grades agree well with the observed thermal runaway behaviors. Overall, this work advances Li‐plating assessment from occurrence identification to the quantitative diagnosis of Li‐plating degree and the corresponding thermal safety grading. The proposed framework provides a basis for Li‐plating risk assessment and safety evaluation of LIBs.

## Graphical abstract

Low N/P cells enable controllable lithium plating, allowing the lithium plating degree (*D*_Li_) to be quantified and linked to thermal safety. Pressure signals during calibration provide a non‐destructive route to diagnose *D*_Li_, while thermal response tests define safety levels and management strategies. The framework remains applicable in commercial 12 Ah cells, supporting practical safety evaluation of lithium‐ion batteries.

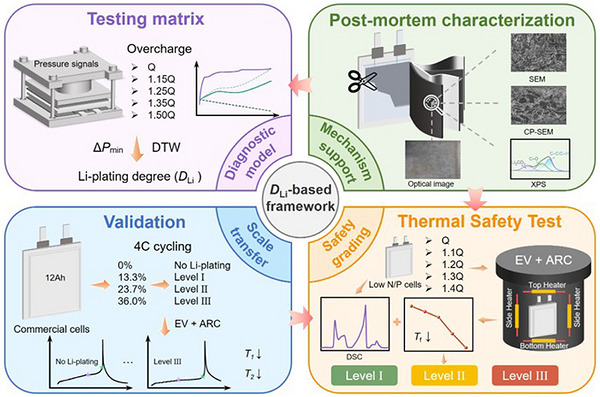

## 1 Introduction

With the growing demand for electric transportation and large‐scale energy storage, lithium‐ion batteries (LIBs) have become a dominant electrochemical energy storage technology [1, 2, 3, 4]. However, fast charging, low temperature operation, and prolonged cycling can induce lithium (Li) plating [5, 6, 7, 8], which accelerates degradation and increases the risk of thermal runaway. Therefore, quantifying the Li‐plating degree and establishing its correlation with thermal safety are critical for the safety assessment of LIBs.

In situ detection techniques are widely regarded as important approaches for real‐time monitoring of Li‐plating evolution. Advanced characterization methods such as nuclear magnetic resonance (NMR) [9, 10], x‐ray diffraction (XRD) [11, 12], and x‐ray computed tomography (XCT) [13, 14] can probe Li deposition in situ while providing detailed structural and microstructural information. However, these techniques generally rely on expensive instrumentation and require stringent testing conditions, which greatly limit their large‐scale industrial application [15, 16]. To improve practical feasibility, methods based on electrochemical signals have been widely used for Li‐plating detection because of their operational simplicity and relatively low cost. Among them, differential voltage analysis (DVA) [17, 18, 19] and differential voltage relaxation (DVR) [20, 21] can provide a quantitative assessment of Li‐plating by monitoring voltage responses and capacity variations. Nevertheless, these methods usually rely strongly on specific testing protocols and characteristic voltage features, which limit their robustness and practical applicability under complex operating conditions. Similarly, electrochemical impedance spectroscopy (EIS) [22, 23, 24, 25] and coulombic efficiency (CE) [26, 27] can provide valuable information for battery degradation analysis, but battery failure often arises from coupled degradation mechanisms, making it difficult for these methods alone to comprehensively and accurately capture Li‐plating evolution. Against this background, techniques that monitor surface expansion stress and thickness variation to reflect internal battery behavior have attracted increasing attention and have shown high sensitivity to abnormalities associated with Li‐plating [28, 29, 30]. Huang et al. [31] achieved high‐precision identification of the critical moment preceding substantial Li deposition by comparing dP/dQ thresholds during charging. Gao et al. [32] further demonstrated the engineering feasibility of pressure signals for passive battery failure detection. Despite the high sensitivity and practical promise of pressure signals, existing studies have mainly focused on Li‐plating identification or early warning. Their use for the quantitative diagnosis of Li‐plating degree and thermal safety grading remains insufficiently explored.

Li‐plating can directly compromise battery thermal safety through its vigorous reaction with the electrolyte, thereby triggering earlier thermal runaway [33]. Li et al. [34] focused on the practical risk posed by Li‐plating and investigated its influence on battery thermal safety under fast charging conditions. They proposed 70°C as a warning temperature for thermal risk associated with Li‐plating during fast charging and pointed out that Li‐plating should be suppressed through optimized cell design and operating strategies. In addition, Zhou et al. [35] further attempted to correlate lithium plating quantification with cell‐level thermal safety characteristics by using plating energy as a quantitative metric and found that the onset temperature of adiabatic self‐exotherm decreased with increasing Li‐plating degree. Although these studies show that Li‐plating affects battery thermal safety, current understanding remains largely limited to thermal responses under specific conditions or individual safety indicators. A systematic quantitative relationship between Li‐plating degree and thermal response that can support risk grading remains lacking.

**FIGURE 1:**
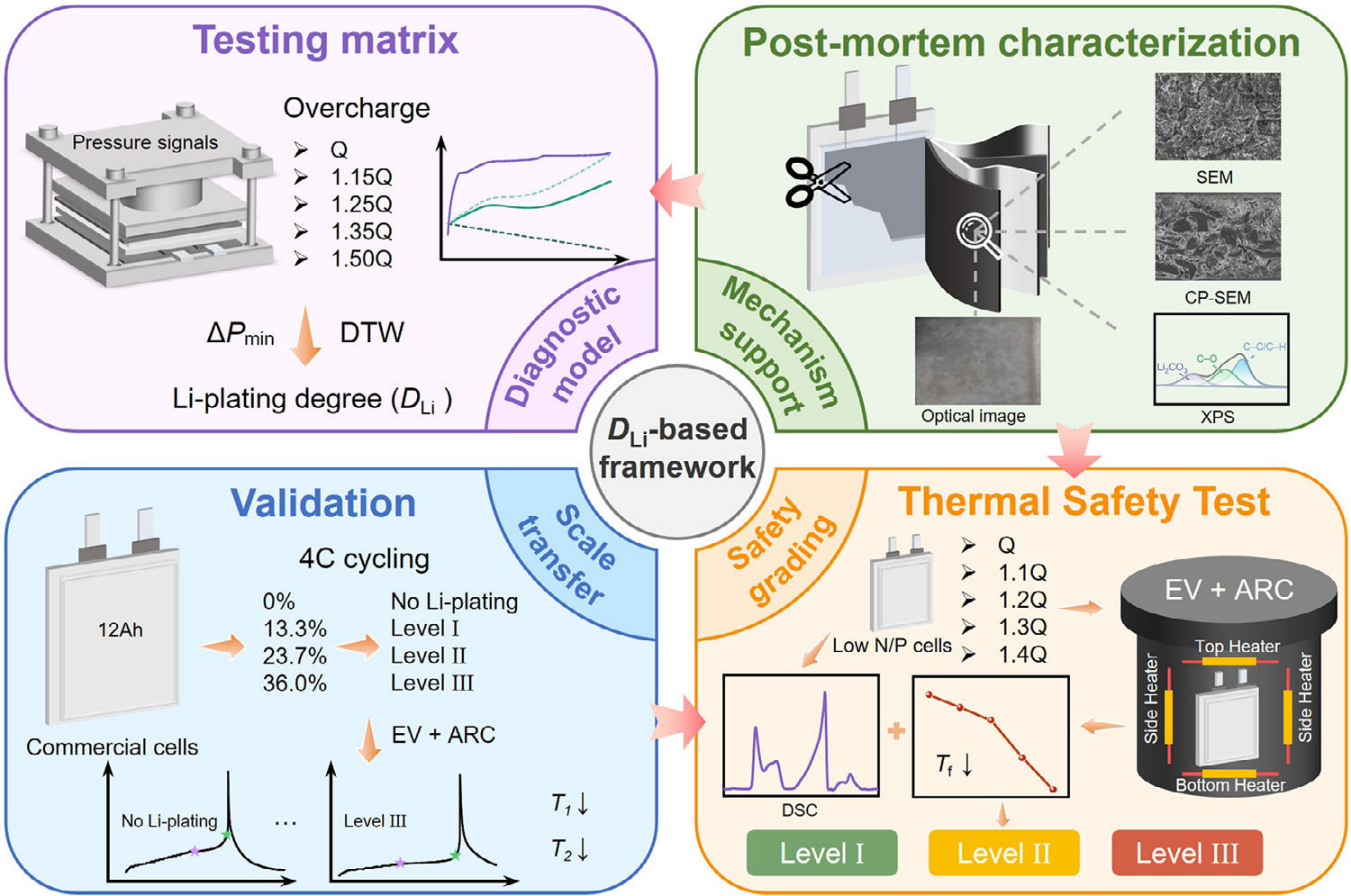
Schematic framework for *D*_Li_ quantification, thermal safety grading, and cross‐scale validation in LIBs.

In this work, low N/P LIBs are constructed by reducing the areal loading of graphite anodes, enabling controllable Li‐plating and establishing a quantitative descriptor of Li‐plating degree, *D*_Li_. Post‐mortem characterizations, including SEM and XPS, are used to reveal the progressive accumulation of Li‐plating and its accompanying reaction products, together with the aggravation of interfacial degradation, thereby clarifying the evolution associated with increasing *D*_Li_. Based on pressure signals collected during calibration cycles, characteristic factors related to Li‐plating are extracted to develop a non‐destructive diagnostic model for *D*_Li_. Accelerating rate calorimeter (ARC) is further employed to establish a quantitative relationship between *D*_Li_ and thermal safety characteristics, from which *D*_Li_ = 20% and 30% are defined as the boundaries for safety grading, thereby delineating three thermal safety levels. Furthermore, cross‐scale validation is conducted in commercial 12 Ah cells to evaluate the transferability of the proposed diagnostic model and grading strategy. Overall, this work establishes a calibration‐based diagnostic model for the quantitative characterization of *D*_Li_ together with a corresponding thermal safety grading and battery management framework, thereby providing a theoretical basis and methodological support for Li‐plating risk assessment, thermal safety evaluation, and graded safety management of LIBs. The schematic framework of this study is shown in Figure 1.

## 2 Results and Analysis

### 2.1 Quantifying Li‐Plating Degree and Electrochemical Analysis

To track the evolution of graphite anode voltage during overcharge, a Li_4_Ti_5_O_12_ (LTO) reference electrode is embedded in the low N/P cell [36]. Figure S2 shows the variation of graphite anode voltage under different charge cutoff capacities. Under the Q condition, the anode voltage gradually decreases with increasing capacity, while its minimum value remains above 0 V vs. Li/Li^+^, indicating that graphite still stays within the voltage range for Li intercalation. In contrast, pronounced differences emerge among the overcharged cells at the end of charge. As shown in the enlarged view highlighted by the gray box, the anode voltage of the overcharged cells crosses 0 V vs. Li/Li^+^ near the charge cutoff and remains below 0 V vs. Li/Li^+^ over a certain capacity interval. It is generally accepted that when the anode voltage falls below 0 V vs. Li/Li^+^, metallic Li deposition becomes thermodynamically favorable, thereby markedly increasing the tendency for Li‐plating [37]. Therefore, the shaded capacity interval in the figure can be regarded as the Li‐plating window of the graphite anode.

As the charge cutoff capacity increases from 1.15Q to 1.50Q, the Li‐plating window progressively widens. Meanwhile, the anode voltage extends further below 0 V vs. Li/Li^+^, indicating that deeper overcharge strengthens the driving force for Li‐plating at the end of charge and prolongs a state prone to Li‐plating.

The cycling performance of low N/P cells under different charge cutoff capacities is shown in Figure S3. The non‐overcharged Q maintains nearly stable capacity within the testing window, while the 1.15Q exhibits only gradual capacity decay. In contrast, the 1.25Q and more severely overcharged groups show markedly accelerated capacity fading, with earlier onset and larger decay as the overcharge depth increases. In particular, the 1.50Q fails by the eighth cycle and cannot continue normal charging. Therefore, although its capacity does not appear to decay to a lower level than that of some other groups in Figure S3, this reflects premature failure rather than improved cycling stability.

**FIGURE 2:**
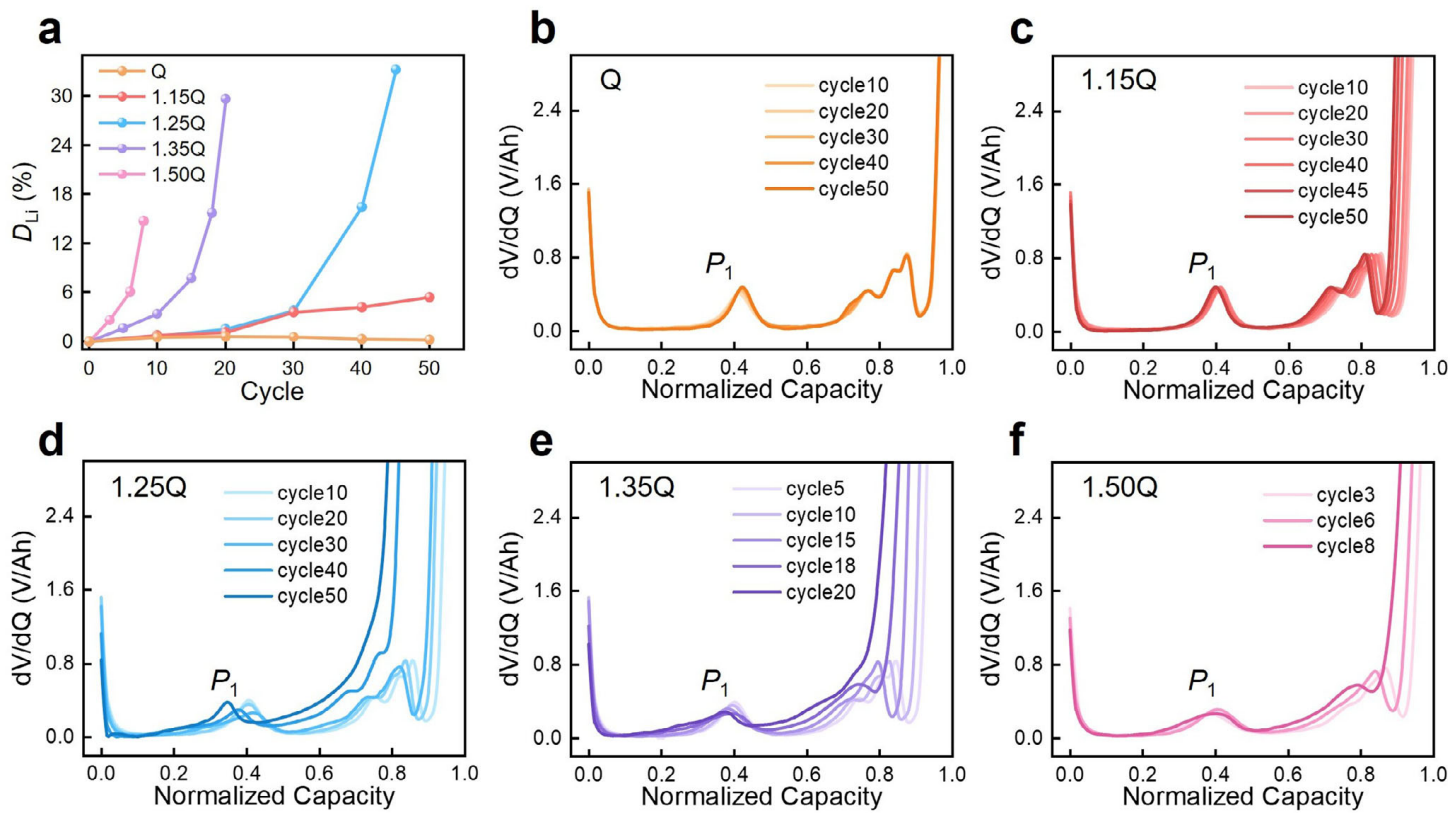
Evolution of *D*_Li_ (a) and dV/dQ curves under different overcharge conditions. (b) Q, (c) 1.15Q, (d) 1.25Q, (e) 1.35Q, (f) 1.50Q.

To quantify the Li‐plating degree under different overcharge conditions, the Li‐plating degree (*D*_Li_) is defined based on the calibration capacity, as shown in Equation (1). Here, *D*_Li_ is not used to represent the mass of metallic Li. Instead, it is a Li‐plating degree index derived from capacity change under controlled overcharge conditions in low N/P cells. This definition is based on the preceding reference electrode results, which show that the overcharged cells enter the Li‐plating regime, as reflected by the anode voltage below 0 V vs. Li/Li^+^ and the progressively widened Li‐plating window. Therefore, the capacity loss used to calculate *D*_Li_ is mainly attributed to Li‐plating and the side reactions induced by plated Li, rather than general aging processes unrelated to Li‐plating. Figure 2a shows the evolution of *D*_Li_ with cycle number under different charge cutoff capacities. Under the Q condition, *D*_Li_ remains close to zero throughout the test window, indicating that no appreciable Li‐plating accumulation is observed. Under the mildly overcharged 1.15Q condition, *D*_Li_ increases gradually and remains below 6%, suggesting that only slight Li‐plating occurs. In contrast, the 1.25Q, 1.35Q, and 1.50Q conditions exhibit distinctly different and much more pronounced evolution patterns. Specifically, *D*_Li_ under the 1.25Q condition increases moderately during the early cycles but rises sharply after 30 cycles and eventually exceeds 30%. Under the 1.35Q condition, *D*_Li_ starts to rise markedly within the first 10 cycles and approaches 30% before failure. By comparison, *D*_Li_ increases rapidly almost from the beginning of cycling and exceeds 15% within a limited number of cycles under the 1.50Q condition.

(1)

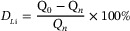

where *Q*_0_ is the first calibration capacity, and *Q_n_* is the *n*‐th calibration capacity.

Figure 2b–f shows the evolution of the dV/dQ curves during cycling under different overcharge conditions, with characteristic peak *P*_1_ taken as the main feature for comparison to elucidate the failure evolution induced by Li‐plating. For the normally cycled Q cell (Figure 2b), both the position and shape of peak *P*_1_ remain largely stable from the initial cycle to the 50th cycle, showing only slight drift. Such gradual variation is more likely associated with reversible early‐cycle equilibration processes within the cell, rather than with any pronounced abnormality or Li‐plating feature [38]. Under the mildly overcharged 1.15Q condition (Figure 2c), peak *P*_1_ shows only a slight leftward shift with limited overall change as cycling proceeds, indicating that mild overcharge exerts only a weak influence on the graphite lithiation phase transition, consistent with the low *D*_Li_ observed for the 1.15Q condition in Figure 2a. When the charge cutoff capacity is increased to 1.25Q (Figure 2d), the migration of peak *P*_1_ becomes much more pronounced and a continuous leftward shift with cycling. This indicates that the progressive accumulation of Li‐plating near the end of charge reduces the cyclable Li inventory and shifts the electrochemical operating window, thereby compressing the characteristic region associated with graphite lithiation toward lower capacity and leading to rapid degradation until failure. Notably, under the more deeply overcharged 1.35Q and 1.50Q conditions, the cells undergo drastic capacity decay and premature failure within fewer cycles in Figure 2e,f. Although peak *P*_1_ still shifts leftward, its migration amplitude does not increase monotonically with overcharge depth and is instead weaker than that in the 1.25Q. This behavior suggests that, under severe overcharge, the large amount of deposited metallic Li and its side‐reaction products may coat or electronically isolate graphite particles at an early stage, causing part of the anode active regions to rapidly lose their effective delithiation capability. Therefore, unlike the sustained migration of *P*_1_ observed in the 1.25Q, the 1.35Q and 1.50Q are more prone to rapid degradation and early failure within a limited number of cycles. The evolution of the dV/dQ curves indicates that different Li‐plating degrees are accompanied by different degrees of electrochemical degradation. The following post‐mortem analysis further explains this relationship from the structural and interfacial perspectives.

### 2.2 Post‐Mortem Analysis and Pressure Signal Evolution Mechanism

**FIGURE 3:**
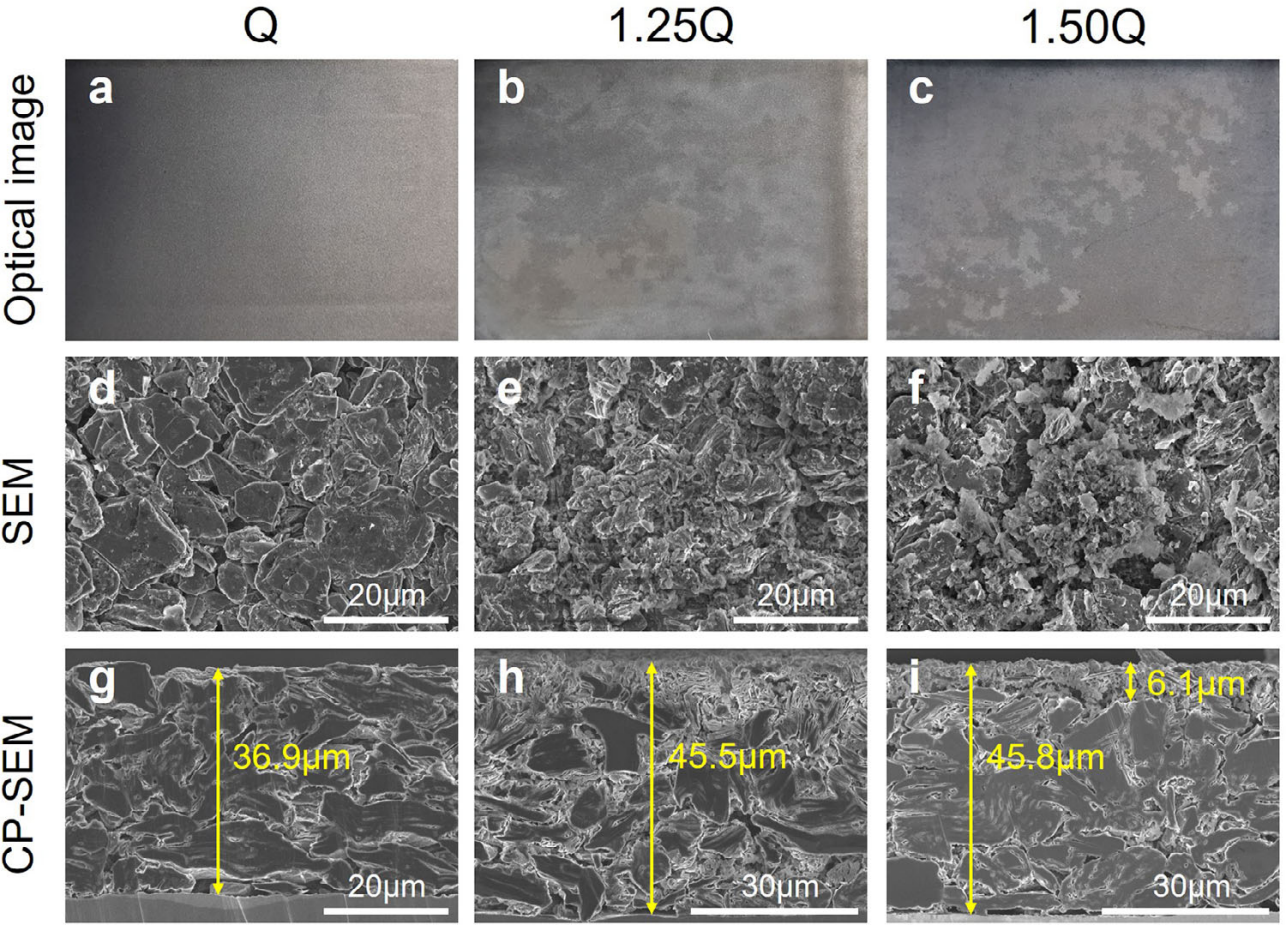
Morphological evolution of graphite anodes. (a–c) Optical images, (d–f) surface SEM, and (g–i) cross‐sectional SEM.

To elucidate the structural and interfacial evolution associated with increasing Li‐plating, the Q‐1.50Q cells after cycling were disassembled and subjected to post‐mortem characterization of microstructure and interfacial chemistry. As shown in Figure 3a–c, compared with the relatively uniform surface morphology of the Q cell, both the 1.25Q and 1.50Q cells exhibit pronounced mottled surface distributions, indicating that Li‐plating and its accompanying side reactions do not proceed in an ideally uniform manner, but are more likely initiated by local polarization differences and progressively amplified during cycling. SEM observations of the electrode surface (Figure 3d–f) further reveal Li‐plating coverage and structural degradation induced by overcharge. Distinct graphite particles are still visible in the Q sample, whereas the 1.25Q sample shows markedly increased surface roughness, blurred particle boundaries, and obvious flocculent coverage, pointing to the gradual occupation of pore channels and active material surfaces by deposited Li and reaction products associated with Li‐plating. When the overcharge depth is further increased to 1.50Q, the surface coverage related to Li‐plating becomes much more pronounced, suggesting that reaction products accumulate rapidly, and structural degradation is already significantly aggravated at an early stage of cycling.

CP‐SEM further verifies, from the perspectives of thickness variation and internal structural evolution, the accumulation of irreversible deformation reflected by the pressure signals (Figure 3g–i). Compared with the Q sample, the anode cross‐sectional thicknesses of the 1.25Q and 1.50Q samples increase by 23% and 24%, respectively. It should be noted that the thickness increase mainly reflects the deposition of metallic Li and associated reaction products, and its contribution to the expansion pressure during calibration is not simply positively correlated. Once the deposited species continuously occupy pore space and weaken effective lithiation pathways, the volume response driven by reversible lithiation and the corresponding working window become compressed. As shown in Figure 3h, obvious particle fracture is observed near the surface region of the 1.25Q sample, intertwined with Li‐plating products, indicating that moderate overcharge over extended cycling more readily induces local stress concentration and particle damage, thereby accelerating pore blockage and aggravating transport limitation. In contrast, the 1.50Q sample exhibits a dense surface layer of about 6.1 µm, dominated by Li‐plating products, without obvious particle fracture. This indicates that under severe overcharge, Li‐plating and side‐reaction products accumulate rapidly at an early stage and dominate the evolution of the interfacial morphology. The electrical isolation effect of this surface layer more directly reduces the effective lithiation area of the active material and aggravates transport limitation, thereby leading to rapid cell failure. The 1.15Q and 1.35Q samples also show progressive changes consistent with those of the 1.25Q and 1.50Q samples in terms of macroscopic surface variation, surface Li‐plating coverage, particle roughening, and cross‐sectional structural evolution (Figure S4).

SEM results of the cathodes and separators for the Q, 1.25Q, and 1.50Q cells further show that, within the cycling window and overcharge conditions adopted in this work, no obvious change is observed in the morphology or particle size of the LFP cathode, and no macroscopic defects such as pore collapse or separator puncture are found (Figure S5). The post‐mortem comparison shows that the major structural and interfacial changes are concentrated on the graphite anode. With increasing overcharge depth, the anode exhibits different degrees of Li‐plating coverage, reaction product deposition, surface roughening, local particle damage, and thickness increase. In contrast, the LFP cathode and separator do not show significant morphological degradation within the same test window. These results indicate that, under the controlled low N/P overcharge conditions, the capacity loss used for *D*_Li_ calculation is mainly dominated by anode Li‐plating and the interfacial reactions induced by plated Li, rather than cathode deterioration or separator damage. These results provide morphological evidence for linking *D*_Li_ with anode Li‐plating and interfacial degradation induced by plated Li under the controlled overcharge conditions.

**FIGURE 4:**
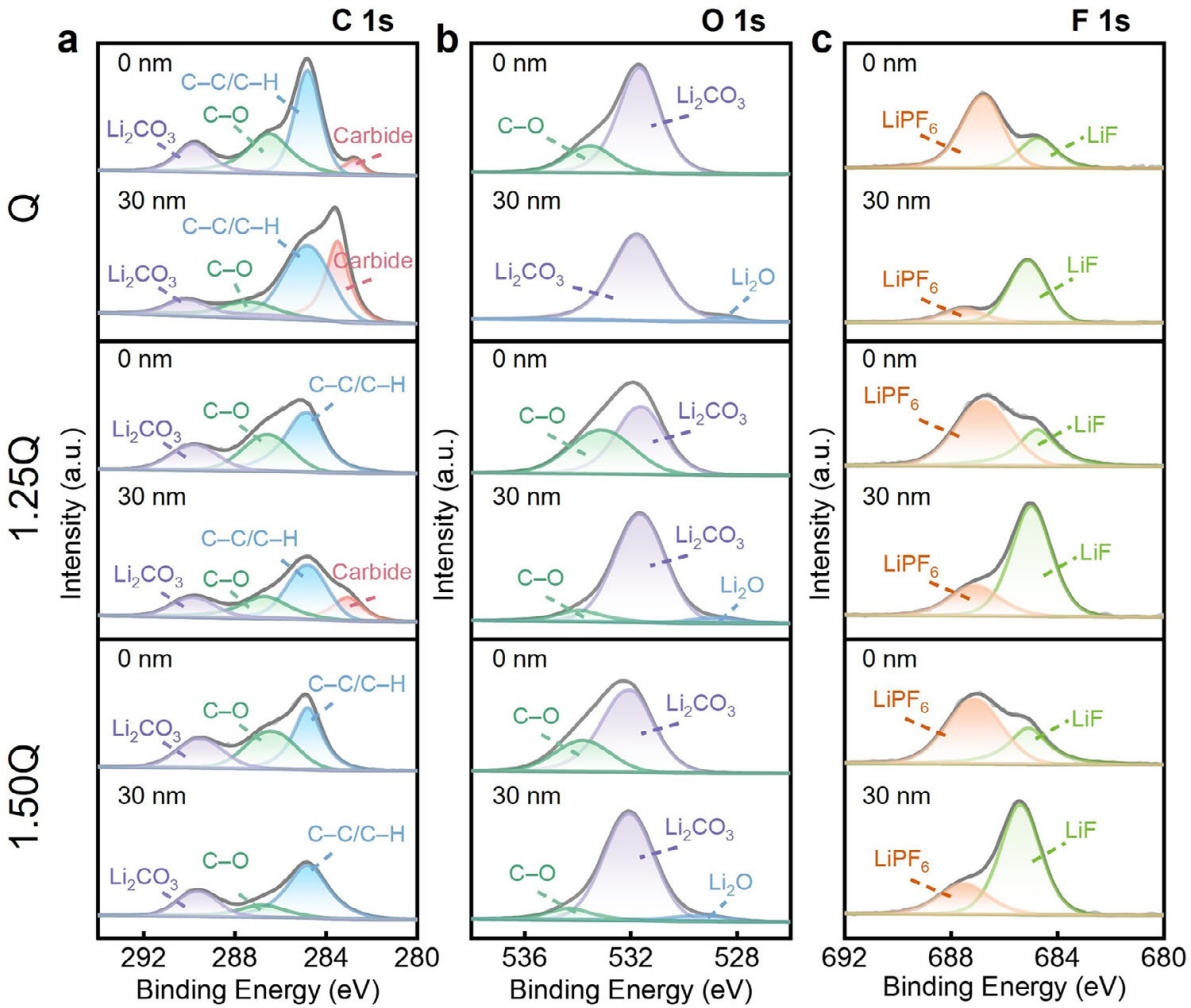
XPS spectra of graphite anodes at 0 and 30 nm depth. (a) Q, (b) 1.25Q, (c) 1.50Q.

To further elucidate the evolution of chemical composition on the graphite anode under different overcharge cycling conditions, XPS is performed on the graphite anodes of Q‐1.50Q cells (Figure 4 and Figure S6). In the C 1s spectra, C─C/C─H, C─O, and Li_2_CO_3_ are observed on the surfaces (0 nm) of all samples, indicating the presence of a hybrid solid electrolyte interphase (SEI) composed of organic and inorganic components. Unlike the 1.25Q sample, the 1.50Q sample does not show a newly emerged carbide peak after etching to a depth of 30 nm. This suggests that the graphite substrate is more readily exposed in the 1.25Q sample after etching, implying a relatively thinner interfacial coverage layer. By contrast, the 1.50Q sample remains dominated by signals from interfacial reaction products after etching, indicating a thicker deposited layer, which is consistent with the dense surface coverage observed in Figure 3. In the O 1s spectra, the overcharged samples exhibit a more pronounced C─O peak at the surface, and an evident C─O signal is still detected after etching to 30 nm, whereas the corresponding C─O peak in the Q sample nearly disappears after etching to 30 nm. This indicates that the oxygen‐containing organic components in the SEI may extend deeper into the interfacial region under overcharge conditions. Considering that repeated Li‐plating and Li‐stripping during overcharge continuously induce SEI rupture, repair, and regeneration, the interphase is more likely to evolve into a chemically complex structure in which organic and inorganic components are intricately mixed. The F 1s spectra further show that the LiF peak is significantly enhanced in the overcharged samples and remains prominent after etching, indicating a stronger tendency for fluorine‐containing inorganic species to enrich in the inner interfacial layer. These results suggest that, with increasing overcharge depth, the interfacial layer on the graphite anode not only becomes thicker overall but also gradually develops a more inorganic‐rich and stratified chemical structure. Together with the morphological evidence above, the XPS results further show that the increase in *D*_Li_ is accompanied by the accumulation of plated Li and reaction products on the anode, as well as the progressive reconstruction of interfacial chemistry. The deposition morphology and relative composition of interfacial products may change at low temperatures or under fast charging. Despite these differences, Li‐plating induced under different conditions is generally accompanied by plated Li accumulation, electrolyte side reactions, and interfacial evolution [6, 39]. These electrochemical and interfacial changes support the use of *D*_Li_ as a Li‐plating degree indicator and show that the low N/P configuration provides a controlled platform for investigating the associated electrochemical, mechanical, and thermal effects.

**FIGURE 5:**
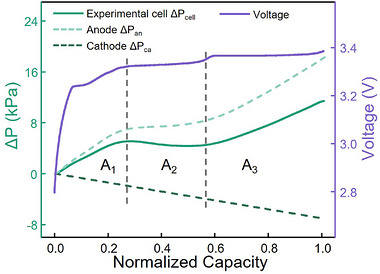
ΔP and voltage evolution with normalized capacity during the calibration process.

Electrode volume changes during LIBs cycling are mainly manifested as pressure responses along the thickness direction. In addition to the reversible volume changes induced by lithiation and delithiation, irreversible processes such as Li‐plating, SEI growth, gas evolution, and structural degradation can also introduce extra deformation, thereby causing cumulative drift of the pressure signal [40]. To establish the correspondence between the pressure signal and electrode volume change, the lattice volume evolution of the graphite anode and LFP cathode at different SOCs is estimated from the x‐ray diffraction (XRD) results [41, 42], as shown in Figure S7. During lithiation, graphite exhibits nonlinear volume expansion behavior, which can be divided into three characteristic regions (A_1_, A_2_, and A_3_). In contrast, the lattice volume change of LFP during delithiation is approximately linear because of its olivine structure and two‐phase transformation mechanism. Based on these lattice volume evolution characteristics and the mechanical decomposition method, Figure 5 presents the evolution of the anode pressure contribution (ΔP_an_), cathode pressure contribution (ΔP_ca_), full cell pressure (ΔP_cell_), and the corresponding voltage profile during the calibration charge process. In region A_1_, ΔP_cell_ increases rapidly with SOC, mainly dominated by the pronounced expansion associated with the initial lithiation of graphite, whereas the shrinkage contribution from LFP remains relatively limited at this stage. Upon entering region A_2_, the growth rate of ΔP_cell_ decreases significantly and even becomes temporarily weakened. This behavior corresponds to the transition of graphite from Stage 2L to Stage 2. During this stage, Li^+^ is mainly rearranged within the existing interlayer galleries rather than continuously opening new interlayer spaces, so its contribution to the increase in interlayer spacing and macroscopic thickness remains limited. Meanwhile, LFP continues to delithiate and contracts approximately linearly. The two effects partially offset each other at the macroscopic level, thereby suppressing the increase in ΔP_cell_ and even causing a local decrease. In region A_3_, graphite undergoes further lithiation and gradually enters a highly lithiated state. As more interlayer galleries are filled with Li^+^, graphite expansion becomes pronounced again and exceeds the shrinkage contribution of LFP, causing ΔP_cell_ to increase monotonically again with further lithiation.

**FIGURE 6:**
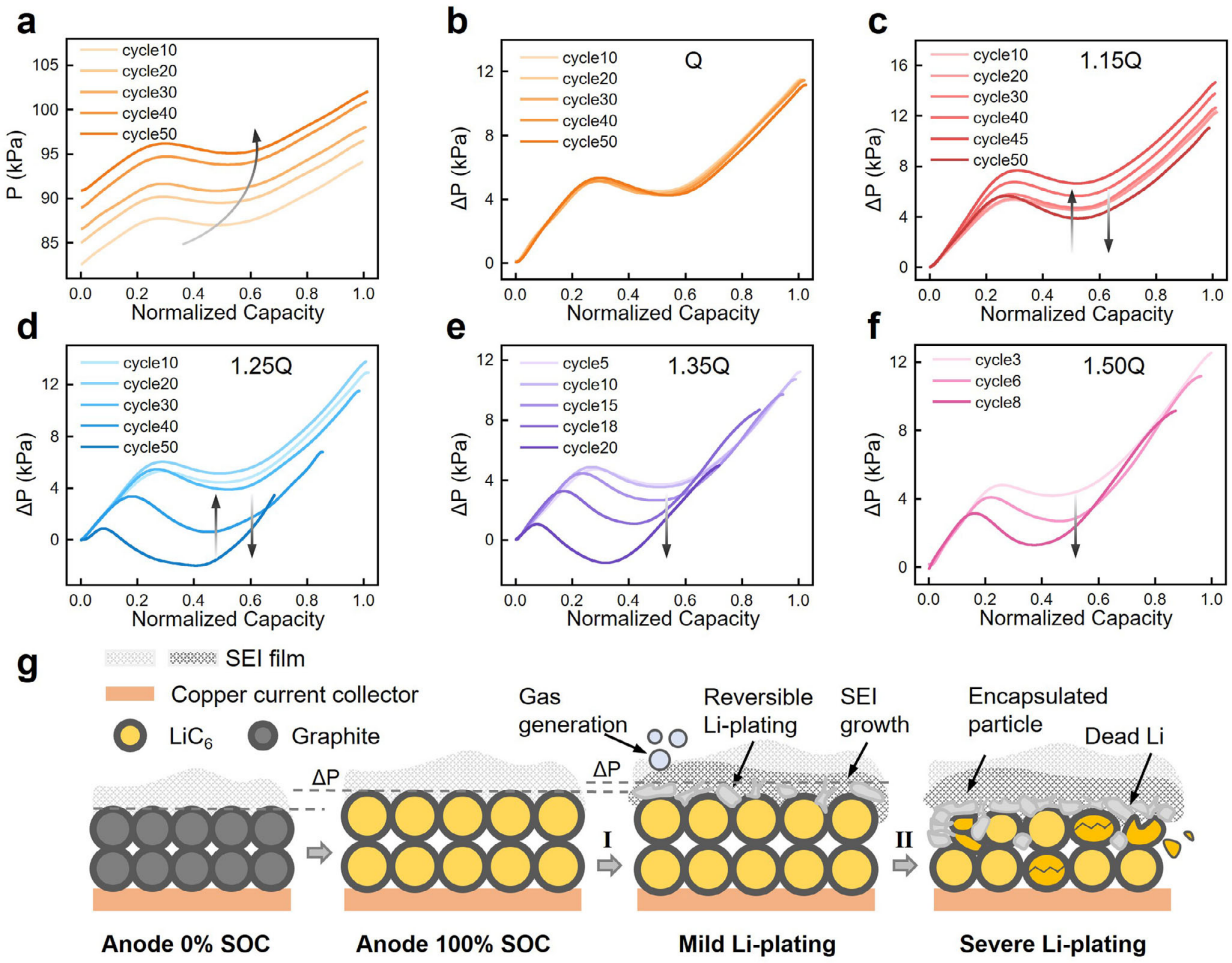
Pressure evolution and its relation to Li‐plating. (a) Pressure curves of the Q cell during calibration at different cycles. ΔP curves of the calibration cycle under (b) Q, (c) 1.15Q, (d) 1.25Q, (e) 1.35Q, and (f) 1.50Q conditions. (g) Schematic illustration of the battery failure mechanism induced by Li‐plating.

As cycling proceeds, the continuous accumulation of irreversible stress causes an overall upward shift in the pressure curves of the cells during the calibration cycles (Figure 6a). To compare the relative expansion behavior during charging across different cycles, the pressure in each calibration cycle is normalized by subtracting its initial value, yielding the relative expansion pressure ΔP referenced to 0 kPa. Figure 6b–f shows the evolution of the charging ΔP curves during the calibration cycles for Q‐1.50Q as the *D*_Li_ progressively increases. Under the Q condition, the overall shape of the ΔP curves remains stable from the first cycle to the 50th cycle, and the segmented features of A_1_‐A_3_ are clearly preserved. Only slight changes are observed in the lengths of these regions and the positions of the turning points along the capacity axis. This gradual variation is consistent with a slight enhancement of reversible lithiation behavior caused by the progressive stabilization and densification of the SEI during mild cycling, and also agrees with the slight capacity increase observed for Q in Figure S3. Because all ΔP curves used for comparison are obtained from calibration cycles without overcharging, their differences reflect the accumulated influence of the preceding cycling history. The stable ΔP profile of the Q cell indicates that short‐term cycling without overcharging induces only limited pressure drift. In contrast, the overcharged cells show pronounced *D*_Li_‐dependent pressure evolution, which is consistent with Li‐plating accumulation generated during the preceding overcharge cycles. Pressure evolution during cycling has been reported to reflect structural and interfacial changes of electrodes [28], indicating its sensitivity to degradation related mechanical evolution. In the overcharged cells studied here, the ΔP evolution occurs concurrently with the anode potential dropping below 0 V vs. Li/Li^+^ and the post‐mortem evidence of degradation caused by Li‐plating on the graphite anode. These results suggest that the extracted pressure features mainly describe mechanical and interfacial evolution associated with Li‐plating under the controlled overcharge conditions.

Under 1.15Q and 1.25Q conditions, the ΔP curves exhibit a two‐stage evolution, characterized by an initial upward shift followed by a downward shift with cycling, which is consistent with the nonlinear accelerated increase in *D*_Li_. In the early overcharge stage I, the overall *D*_Li_ remains low, and its growth rate is still relatively slow. Relative to the first cycle baseline, the ΔP curves shift upward as a whole, accompanied by an increased slope in region A_1_ and a slight contraction of regions A_1_ and A_2_ along the capacity axis. This behavior mainly originates from the formation of a small amount of deposited Li on the graphite surface during the initial overcharge cycles, which occupies part of the lithiation pathways in advance and thereby increases local stress. Meanwhile, the periodic expansion and contraction of the graphite lattice continuously exposes fresh surfaces that keep reacting with the electrolyte, leading to repeated rupture, repair, and regeneration of the SEI. As a result, part of the reversible Li inventory is consumed, accompanied by changes in Li‐insertion and diffusion behavior. The continuous evolution of the interfacial structure also increases the sensitivity of the cell to volume change. The combined effect of these factors leads to an overall upward displacement of the ΔP curves and an increased slope in region A_1_ during stage I, thereby laying the foundation for more severe interfacial and structural degradation in the subsequent cycles.

When cycling enters stage II, the ΔP curves gradually shift downward and contract markedly along the capacity axis, manifested by a decrease in the slope of region A_1_ and an increase in the absolute slope of region A_2_ (Figure 6c,d). This transition is consistent with the continued accumulation of Li‐plating together with the significant aggravation of interfacial degradation and structural damage. Deposited Li continuously accumulates on the anode surface and gradually covers graphite particles. Together with local stress concentration caused by repeated volume change under overcharge conditions, this process further triggers graphite particle fracture and structural collapse, resulting in the loss of effective lithiation pathways and active material. Previous studies have shown that a typical SEI can be regarded as a composite structure consisting of a dense inorganic inner layer and a loose organic outer layer [43]. The outer layer is more permeable to the electrolyte and has an effective Young's modulus markedly lower than that of lithiated graphite [44, 45]. Under overcharge conditions, continuous SEI growth and structural loosening accelerate Li inventory loss and aggravate interfacial instability. At the same time, they reduce the overall mechanical stiffness of the anode, leading to a progressive decrease in the slope of region A_1_ with increasing *D*_Li_. As diffusion pathways become blocked and the effective reaction area decreases, the graphite lithiation phase transition becomes increasingly incomplete, and the associated macroscopic volume expansion is suppressed. In contrast, the shrinkage of LFP during delithiation remains relatively stable, thereby amplifying the response mismatch between anode expansion and cathode contraction and leading to a continuous increase in the absolute slope of region A_2_. Under 1.25Q condition, the slope of region A_3_ increases further, indicating that deposited Li has already accumulated substantially during the preceding cycles, thereby further blocking the effective diffusion pathways of Li^+^ and restricting their transport. As the diffusion resistance continues to increase, the pressure increment during the late stage of charging is markedly amplified even at relatively low current density.

For the 1.35Q and 1.50Q overcharge conditions (Figure 6e,f), the deeper overcharge causes rapid performance deterioration at an early stage and drives the cells directly into a failure state associated with severe Li‐plating. As a result, their ΔP curves exhibit features similar to those observed in stage II of the 1.25Q condition from the onset of cycling. At this stage, a large amount of deposited Li already exists on the graphite surface, covering and isolating a considerable fraction of graphite particles and severely blocking Li^+^ transport pathways. As diffusion limitation becomes increasingly severe, the ΔP curves continue to contract, and the absolute slopes of regions A_2_ and A_3_ further increase, indicating that structural degradation and transport hindrance during lithiation have developed into a more serious failure stage.

Figure 6g summarizes the coupled effects of Li‐plating, SEI evolution, and structural degradation on the pressure response. As the cells evolve from a non‐plating condition to mild and then severe Li‐plating, the pressure response of graphite particles gradually changes from being dominated by reversible volume variation during lithiation to being governed by irreversible deformation accumulation and blockage of Li^+^ transport channels. This transition is further accompanied by dead Li formation, gas evolution, and SEI thickening, which together drive the continuous deviation of the mechanical state of the cell.

### 2.3 Non‐Destructive Diagnostic Model for *D*_Li_ Based on Pressure Signals

**FIGURE 7:**
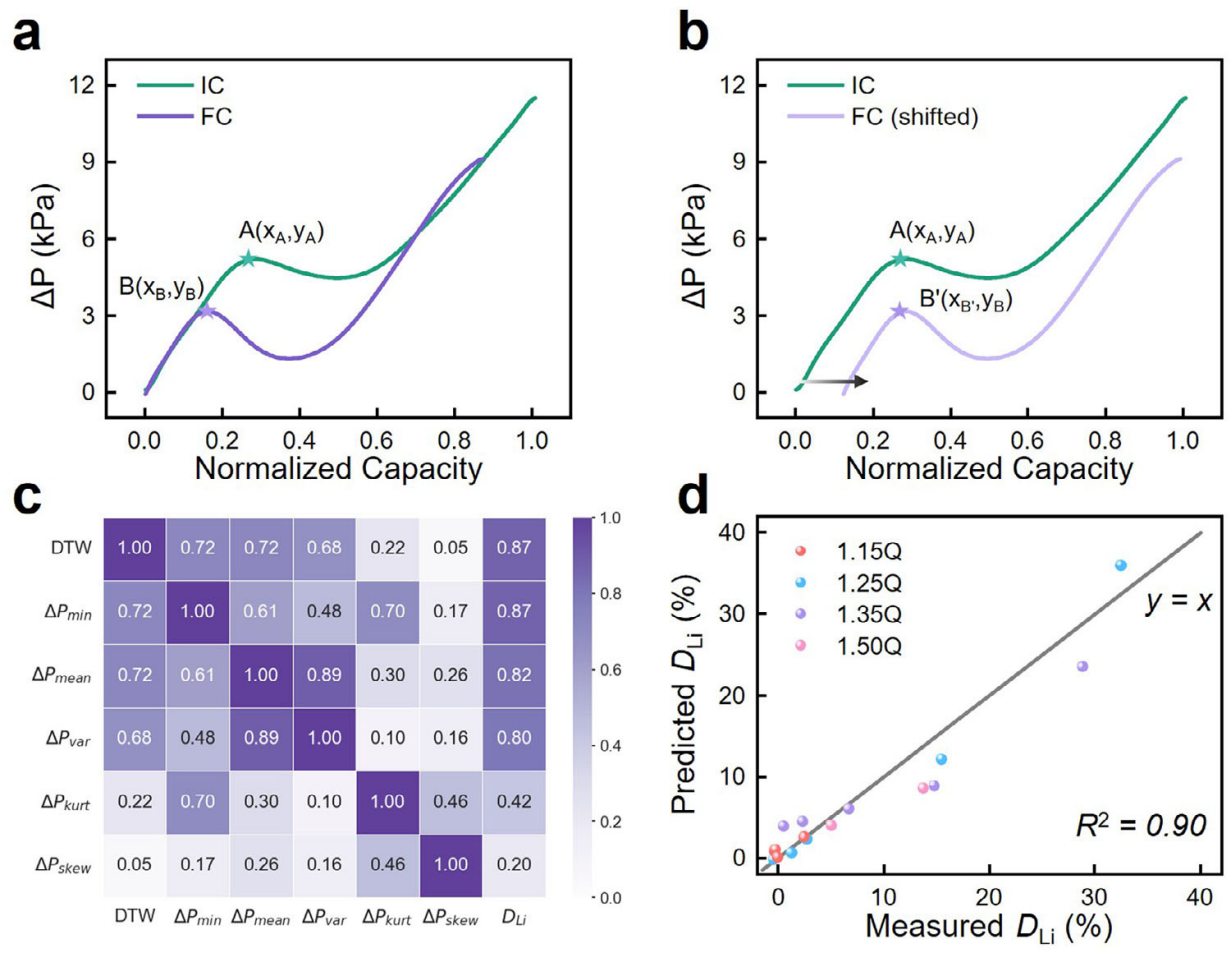
Preprocessing and feature analysis of pressure signals for *D*_Li_ prediction. (a) ΔP curves under IC and FC conditions. (b) Preprocessed ΔP curves and characteristic points. (c) Correlation heatmap between pressure features and *D*_Li_. (d) Relationship between predicted and measured *D*_Li_.

To extract the pressure response associated with Li‐plating accumulation and enable quantitative diagnosis of *D*_Li_, a pressure signal decoupling and feature extraction method is developed. As shown in Figure S8, the Initial Cycle (IC) represents the pressure baseline of the cell during the first calibration charge, whereas the Final Cycle (FC) denotes the pressure curve of the same cell during calibration charging after cycling. Compared with the IC, the FC shows an overall upward shift together with a certain degree of contraction, reflecting the combined evolution of accumulated stress and reversible volume response. The relative expansion pressure ΔP (Figure 7a,b) is adopted to characterize the pressure response during lithiation and delithiation in the current calibration cycle, while retaining the accumulated deformation relative to the initial state.

Under overcharge conditions, the accumulated Li‐plating and aggravated anode polarization are manifested as the contraction of Δ*P*_FC_ relative to Δ*P*_IC_ in the pressure curves. To compare the difference between the two curves within the same phase‐transition stage, the horizontal axis of Δ*P*_FC_ is translated as a whole to align the key knee points (Equation 2). On this basis, the aligned curves are further calculated (Equation 3) to reduce the contribution from normal reversible lithiation and delithiation, while highlighting the residual pressure variation associated with Li‐plating accumulation and its coupled interfacial evolution. This treatment further reduces the influence of baseline offset, pressure drift caused by cycling, and slowly varying pressure contributions that may arise from gas generation. The resulting decoupled curves improve comparability across cycles and samples and increase the correlation between the extracted features and *D*_Li_. Under the present experimental conditions, Li‐plating is the dominant degradation process. Therefore, the extracted features mainly reflect the decoupled pressure response associated with accumulated Li‐plating and its coupled interfacial evolution, rather than the overall pressure evolution.

(2) $$\Delta P_{\mathrm{FC}}^{\prime }(x)=\Delta P_{\mathrm{FC}}\left(x+\left(x_{A}-x_{B}\right)\right)$$

(3)

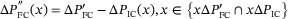

where *x_A_* and *x_B_* denote the positions of the knee points corresponding to the transition from region A_1_ to region A_2_ on the Δ*P*_IC_ and Δ*P*_FC_ curves along the normalized capacity axis. $x\Delta P_{\mathrm{FC}}^{\prime }$ and *x*Δ*P*_IC_ denote the ranges of normalized capacity covered by the $\Delta P_{\mathrm{FC}}^{\prime }$ and Δ*P*_IC_ curves.

After the 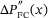 curves are obtained, statistical features that describe the amplitude and distribution of the differential pressure response are further extracted, including Δ*P*_min_, Δ*P*_mean_, Δ*P*_var_, Δ*P*_kurt_, and Δ*P*_skew_. These features, together with dynamic time warping (DTW), are treated as candidate features for correlation analysis to evaluate their relevance to *D*_Li_ (Figure 7c). Among these candidate features, DTW is used to evaluate the overall profile difference of the differential pressure curve relative to the initial baseline curve while retaining the capacity‐dependent sequence information of the pressure response. Meanwhile, DTW shows a strong correlation with *D*_Li_, and is therefore selected as one of the input features for model construction. Its algorithm schematic and detailed calculation procedure are provided in Note S1 and Figure S9. In contrast, Δ*P*_min_, Δ*P*_mean_, Δ*P*_var_, Δ*P*_kurt_, and Δ*P*_skew_ are statistical descriptors derived from the aligned differential pressure response. Although they are calculated from the whole curve, they mainly describe statistical characteristics such as the minimum value, average value, dispersion, and distribution shape of pressure differences, and do not retain the profile evolution along the capacity axis. Considering the limited number of samples, the diagnostic model is trained using two input features to balance prediction accuracy and physical interpretability, while reducing the risk of overfitting caused by introducing too many features. Among the ΔP‐derived descriptors, Δ*P*_min_ reflects the most pronounced local pressure contraction after curve alignment and shows a strong correlation with *D*_Li_. Compared with Δ*P*_mean_ and Δ*P*_var_, Δ*P*_min_ has a higher correlation with *D*_Li_ and a more direct physical meaning related to local pressure contraction. Although Δ*P*_min_ shows a higher correlation with DTW than Δ*P*_var_ does, the comparison of different two‐feature combinations in Table S2 shows that “DTW + Δ*P*_min_” gives better fitting performance than the other tested feature inputs. Therefore, DTW and Δ*P*_min_ are selected as the input features for model construction, capturing both curve‐shape evolution and representative local pressure contraction. To further establish a diagnostic model transferable to other types of cells, the feature Δ*P*_min_ is normalized. As shown in Figure 6, Δ*P*_min_ is located close to the knee point between regions A_2_ and A_3_. Accordingly, the reference relative pressure, Δ*P*_ref_, is defined as the mean ΔP value at this transition obtained from three baseline cells tested under the Q condition without overcharge using the C/10 calibration protocol. The model uses the dimensionless ratio Δ*P*_min_/Δ*P*_ref_, rather than the absolute value of Δ*P*_min_. For cells of the same format, Δ*P*_ref_ is used as a fixed normalization parameter. When the cell format changes, it is redetermined from the corresponding baseline cells under the same calibration conditions. Using DTW and the normalized Δ*P*_min_ as the two input features, a non‐destructive diagnostic model for *D*_Li_ is established (Equation 4).

(4) $$D_{Li}=4.18+0.025\times DTW-11.23\times \frac{\Delta P_{\min}}{\Delta P_{ref}}$$

Figure 7d compares the predicted *D*_Li_ with the measured *D*_Li_. The data points are generally distributed close to the reference line *y* = *x*, indicating good agreement and low prediction error (R^2^ = 0.90, RMSE = 3.02%, and MAE = 2.49%). These results demonstrate that the combination of DTW and normalized Δ*P*_min_, extracted from pressure signals, enables effective quantitative characterization of *D*_Li_ across different overcharge depths. The two input features of the model also possess clear and complementary physical significance. DTW captures the global profile difference caused by the shift of the electrode operating window and the compression of phase‐transition intervals, whereas Δ*P*_min_ reflects the lower bound change of the local mechanical response induced by accumulated Li‐plating. These two features jointly improve the discrimination capability of the model for cells at different degradation stages and with different *D*_Li_. From a computational perspective, the proposed diagnostic model involves only the extraction of two one‐dimensional pressure features followed by evaluation using a fixed linear equation. Both DTW and Δ*P*_min_ are obtained from the pressure curves collected during the C/10 calibration cycles, rather than being calculated continuously during battery operation. These characteristics keep the hardware requirements low and support the implementation of the model as a periodic diagnostic module for embedded battery management system (BMS) applications.

### 2.4 Thermal Response Behavior and Safety Grading

To define the thermal safety boundaries of cells with different *D*_Li_, five fresh cells with N/p = 0.65 are charged at 1C to Q, 1.1Q, 1.2Q, 1.3Q, and 1.4Q, respectively, and then subjected to ARC tests. To avoid equating the onset of self‐heating detected by ARC with the true onset temperature of a single reaction, *T*_f_ is defined here as the temperature at which the cell is first detected as self‐heating under the ARC criterion and enters the adiabatic tracking stage, namely the first detectable self‐heating temperature.

**FIGURE 8:**
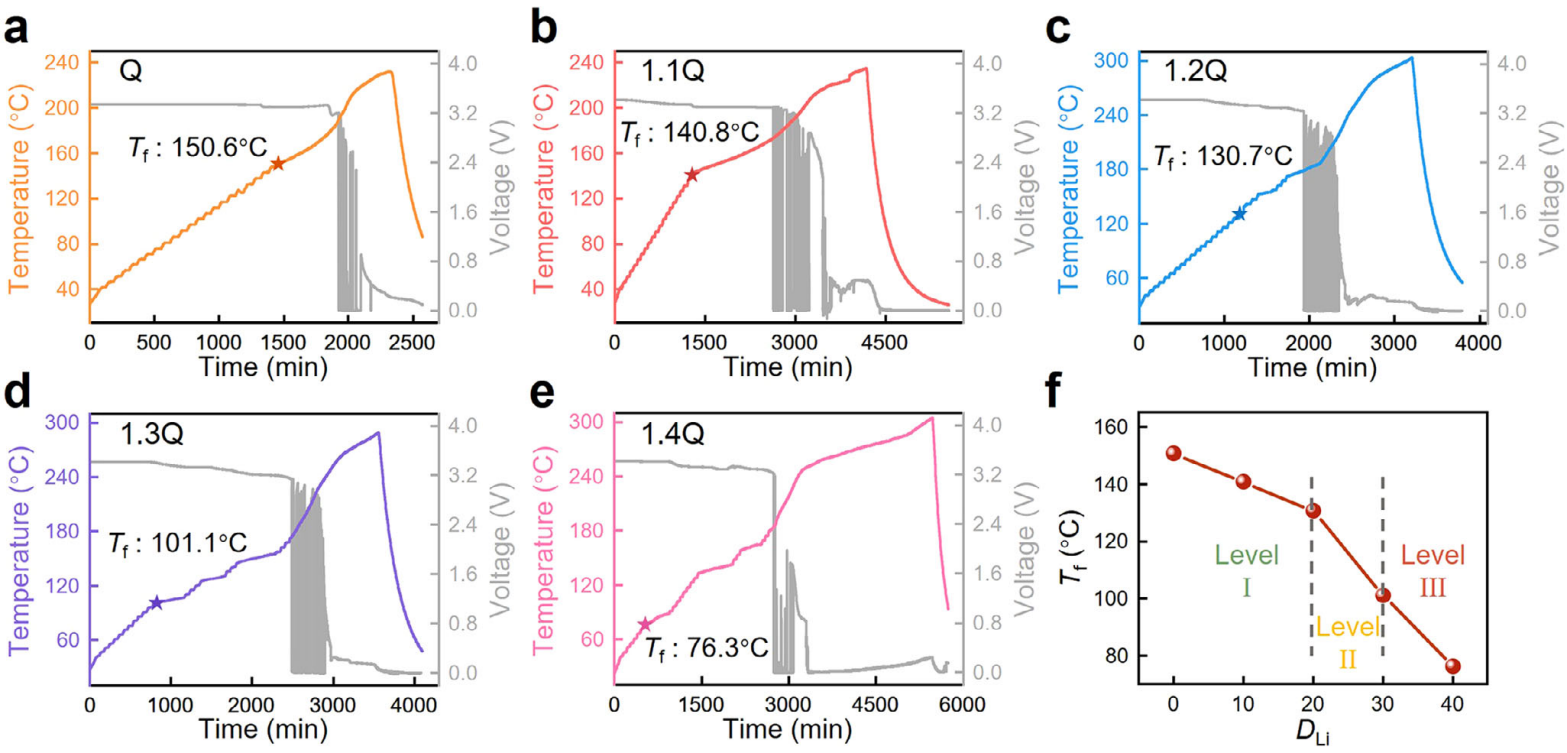
Thermal response evolution with increasing *D*_Li_. (a–e) ARC tests of cells with different *D*_Li_ levels. (f) Segmented relationship between *T*_f_ and *D*_Li_, with the corresponding thermal safety levels delineated by the boundaries at *D*_Li_ = 20% and *D*_Li_ = 30%.

As shown in Figure 8, some highly overcharged cells satisfy the adiabatic criterion more than once during heating and repeatedly re‐enter the adiabatic state. With increasing overcharge depth, *T*_f_ decreases monotonically, indicating that Li‐plating markedly lowers the threshold for entering a detectably self‐heating state. It should be emphasized that *T*_f_ is not identical to the true onset temperature of the earliest exothermic reaction inside the cell. It is more appropriately understood as the temperature at which the overall heat generation rate, arising from the superposition of multiple exothermic processes, first exceeds the ARC trigger criterion of 0.02°C/min. In the Q cell, *T*_f_ appears at 150.6°C, whereas DSC detects exothermic signals at lower temperatures (Figure S10a), indicating that slow exothermic reactions may already occur before *T*_f_ is reached, but their heat generation rates remain below the ARC trigger criterion and therefore cannot be identified in a stable and reproducible manner. Although lowering the trigger criterion would in principle allow earlier detection of weak heat release, it would also amplify the influence of baseline drift and residual heat loss on the calculated heat generation rate, thereby reducing comparability between cells. Therefore, *T*_f_ is treated here as an integrated threshold for detectable self‐heating and is used for comparison among cells, rather than being interpreted as the true onset temperature of any single reaction. By contrast, the overcharged cells show much lower *T*_f_ values and are detected earlier. This can be attributed to Li‐plating and its highly reactive interfacial products, which undergo a series of exothermic reactions with the electrolyte and promote continuous SEI reconstruction and thickening, making it easier for the heat generation rate to exceed the ARC criterion. Furthermore, as the overcharge depth increases, the cells trigger self‐heating multiple times and repeatedly enter the adiabatic state during heating (Figure 8b–e). This indicates that the thermal response is not governed by a single dominant process, but more likely results from interfacial reactions associated with Li‐plating and local short circuits that are activated sequentially over different temperature ranges, leading to a staged superposition of exothermic events.

In Figure 8a, the Q cell shows a slight voltage perturbation in the range of 140°C–150°C and then reaches *T*_f_ at 150.6°C. This temperature range coincides with the characteristic melting temperature of the separator used in this work (Figure S10b) [46]. The slight voltage disturbance in this range may be associated with micro‐short circuits induced by changes in the microporous structure of the separator. As the temperature increases further, SEI decomposition begins and exposes fresh reactive interfaces. Side reactions between the electrode and electrolyte are thereby intensified and release heat, eventually driving the overall heat generation rate beyond the ARC criterion. By contrast, the *T*_f_ of the 1.1Q cell decreases to 140.8°C, which nearly coincides with the characteristic melting range of the separator. This can be attributed to the deeper lithiation state and stronger reducing ability of the anode under the 1.1Q overcharge condition. Once the relatively unstable organic components in the SEI start to decompose thermally, reduction reactions between highly lithiated graphite and the electrolyte can occur earlier, thereby causing a decrease in *T*_f_. Because the amount of plated Li remains limited, the thermal response still largely retains the characteristics associated with separator melting and conventional interfacial side reactions, resulting in only a slight increase in thermal risk.

When the overcharge depth is further increased to 1.2Q, 1.3Q, and 1.4Q (Figure 8c–e), the cells exhibit multiple self‐heating stages during heating, indicating the presence of several exothermic sources that are activated sequentially with increasing temperature. Taking the 1.4Q cell as an example, its *T*_f_ appears at 76.3°C, far below the separator melting range, and is therefore more likely associated with severe interfacial instability caused by Li‐plating. Early reactions between deposited Li and the electrolyte can trigger heat release at relatively low temperature and are accompanied by a slight voltage decrease. Because the heat released at this stage remains limited, the cell temporarily exits the adiabatic criterion. As the temperature subsequently approaches the separator melting range, micro‐short circuits and local hot spots become more likely to form. The resulting heat release, together with the heat generated by interfacial reactions involving plated Li, further increases the overall heat generation rate. A later self‐heating event is therefore triggered at 131.2°C, accompanied by a pronounced voltage perturbation. The Al_2_O_3_ coating improves the thermal stability of the separator and partially suppresses its shrinkage, which may account for the transient voltage rebound or fluctuation. With further heating, side reactions between the electrodes and the electrolyte intensify, while the separator structure continues to deteriorate at high temperature, eventually causing larger‐scale internal short circuits and a sharp voltage drop. Compared with 1.4Q, the *D*_Li_ in 1.2Q and 1.3Q is lower, and their *T*_f_ values rise to 130.7°C and 101.1°C, respectively. In particular, the 1.2Q cell remains in the self‐heating state for a relatively short duration after *T*_f_, and the characteristic temperature range of the subsequent self‐heating event is similar to that of the 1.1Q cell. This similarity suggests that the overall thermal response pattern remains close to that under mild Li‐plating, and the further decrease in Tf indicates an increasing contribution from reactions involving plated Li to detectable self‐heating. The 1.2Q condition, therefore, represents a transitional state between the limited change observed at 1.1Q and the markedly earlier self‐heating observed at higher *D*_Li_. For the 1.3Q cell, *T*_f_ decreases sharply to 101.1°C, well below the separator melting range observed for this cell type. This suggests that low temperature interfacial reactions associated with plated Li have become a major contributor to the early detectable self‐heating response. Overall, as Li‐plating accumulates, the thermal response evolves from a single trigger at high temperature to a multistage process. Interfacial reactions involving plated Li and SEI destabilization can occur progressively earlier during heating and may be amplified by local hot spots or internal short circuits, leading to earlier and repeated self‐heating events.

On this basis, the relationship between *D*_Li_ and battery thermal safety is further established in this work. As shown in Figure 8f, *T*_f_ decreases with increasing *D*_Li_ and exhibits a segmented trend, indicating that Li‐plating accumulation progressively reduces the thermal safety margin of the cell. When *D*_Li_ increases from 0% to 20%, *T*_f_ gradually decreases from 150.6°C to 130.7°C, corresponding to an average decrease of approximately 1.0°C for each 1% increase in *D*_Li_. Within this range, the thermal response still retains characteristics associated with separator melting and conventional interfacial side reactions, and the decrease in *T*_f_ remains relatively limited. When *D*_Li_ increases from 20% to 30%, *T*_f_ decreases sharply to 101.1°C, and the average rate of decrease increases to approximately 3.0°C for each 1% increase in *D*_Li_. This accelerated decline indicates an increasing contribution from reactions involving plated Li to the early detectable self‐heating response. When *D*_Li_ reaches or exceeds 30%, *T*_f_ falls within a distinctly lower range of approximately 76°C–101°C, indicating that detectable self‐heating can be triggered at substantially lower temperatures. The boundary at *D*_Li_ = 20%, therefore, corresponds to the transition from a gradual to an accelerated decrease in *T*_f_, whereas *D*_Li_ = 30% marks the transition to a distinctly lower *T*_f_ range with markedly increased thermal risk. Based on these two changes in thermal response, *D*_Li_ = 20% and *D*_Li_ = 30% are selected as the boundaries for safety grading, thereby delineating three thermal safety levels. It is worth noting that related literature [47] has also reported that when the amount of Li‐plating is controlled within approximately 25% of the lithiated graphite capacity, the cell can still maintain relatively acceptable safety performance. This is close to the warning threshold on the order of 20% derived here from the consequence mapping between *T*_f_ and *D*_Li_. From a more conservative risk management perspective, 20% is therefore selected in this work as the management boundary for triggering early intervention. The N/p = 0.65 cells are used in this work to construct controllable Li‐plating states and establish the relationship between *D*_Li_ and thermal response, rather than to limit the proposed grading framework to this specific N/P ratio. Different N/P ratios may influence Li‐plating formation and accumulation, thereby affecting the quantitative boundaries of the grading framework. A larger anode capacity margin may require more severe charging conditions, lower temperatures, or longer cycling to reach comparable Li‐plating accumulation, whereas a smaller anode margin can promote an earlier transition into degradation dominated by Li‐plating. In addition, changes in electrode balance may alter the spatial distribution of plated Li and the extent of accompanying side reactions, thereby shifting the correspondence between *D*_Li_ and thermal response. Therefore, the numerical boundaries of *D*_Li_ = 20% and *D*_Li_ = 30% should be regarded as reference thresholds derived from the present LFP/graphite system and safety criterion, rather than strictly universal values. At the same time, because the grading framework is established based on the relationship between *D*_Li_ and thermal response rather than the N/P ratio itself, the same safety evaluation concept can be extended to cells with normal N/P designs once Li‐plating occurs and *D*_Li_ can be evaluated, with necessary calibration of the specific thresholds.

### 2.5 Cross‐Scale Validation and *D*_Li_‐Based Safety Evaluation

To examine whether the pressure‐based *D*_Li_ diagnostic model and the three thermal safety levels identified above can be extended beyond the designed low N/P system, four commercial 12 Ah LFP pouch cells with identical specifications are employed for validation. Different from the low N/P cells used for controllable Li‐plating construction, the commercial cells have a practical electrode balance and develop Li‐plating during 4C cycling. The larger cell capacity and different Li‐plating induction pathway broaden the validation conditions and enable further evaluation of the feasibility of the proposed diagnostic model and safety grading framework. These cells are denoted as CV_1, CV_2, CV_3, and CV_4. CV_1 serves as the baseline cell and is subjected only to C/10 calibration. To construct degradation samples dominated by Li‐plating, CV_2, CV_3, and CV_4 are subjected to 4C cycling for 5, 10, and 30 cycles, respectively, under conditions consistent with those used for the low N/P cells. During the test, C/10 calibration cycles are inserted after the 4C cycling, and *D*_Li_ diagnosis is performed using the pressure curves collected during these calibration cycles. In this design, the 4C cycling is used to induce Li‐plating accumulation, whereas the pressure‐based diagnosis is conducted during the subsequent calibration process. Based on the *D*_Li_ diagnostic model established in Section 2.3, features are extracted from the calibration pressure curves of CV_2, CV_3, and CV_4. Using the same C/10 calibration protocol, three fresh commercial 12 Ah cells are used as baseline cells to redetermine Δ*P*_ref_, which is then used to normalize Δ*P*_min_. Before calculating the DTW feature, the pressure curves from the initial and final calibration cycles are separately subjected to Z‐score standardization following the procedure described in Note S1. The calculated *D*_Li_ values after 4C cycling are 13.3%, 23.7%, and 36.0%. According to the three thermal safety levels identified in Section 2.4, these three cells are classified as Level I (low risk), Level II (moderate risk), and Level III (high risk), respectively.

**FIGURE 9:**
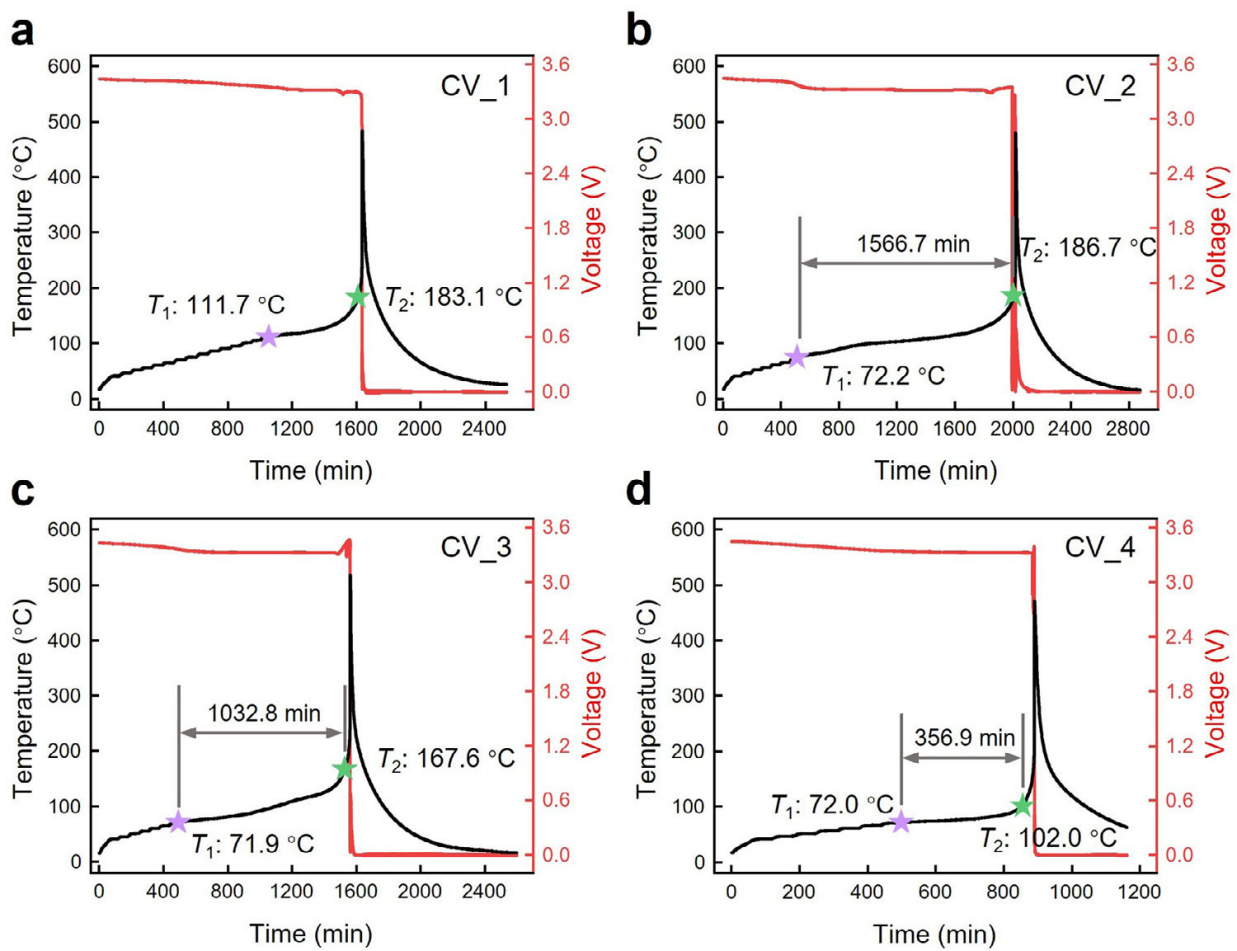
Cross‐scale validation by ARC tests of cells with different *D*_Li_ levels. (a) CV_1, (b) CV_2, (c) CV_3, (d) CV_4.

To further examine the consistency between the predicted safety grades and the measured thermal responses, ARC tests are performed on CV_1, CV_2, CV_3, and CV_4 under the same ARC criterion. Here, the self‐heating temperature (*T*_1_) is defined as the temperature at which the heating rate reaches 0.02°C/min, whereas the thermal runaway triggering temperature (*T*_2_) is defined as the temperature at which the heating rate reaches 1°C/s. As shown in Figure 9, all validation cells exhibit typical thermal runaway evolution during heating, and increasing *D*_Li_ reduces cell thermal stability, consistent with the thermal degradation trend observed in the low N/P cells. For the baseline cell CV_1, *T*_1_ and *T*_2_ are 111.7°C and 183.1°C, respectively. By contrast, after 4C fast charging cycling, the *T*_1_ values of CV_2, CV_3, and CV_4 all decrease to about 72°C, indicating that Li‐plating causes the cells to enter a detectably self‐heating state at a lower temperature and significantly weakens their early stage thermal stability. However, the *T*_1_ values of the three Li‐plating cells remain close to each other, suggesting that for commercial 12 Ah cells, *T*_1_ mainly reflects whether low temperature side reactions associated with Li‐plating have been activated, whereas its ability to further distinguish *D*_Li_ is relatively limited.

By contrast, *T*_2_ exhibits much stronger discrimination among different *D*_Li_. The *T*_2_ of CV_2 is 186.7°C, very close to that of the baseline cell CV_1, indicating that although mild Li‐plating can trigger early self‐heating in advance, it does not yet significantly lower the threshold for thermal runaway triggering. As Li‐plating becomes more severe, the *T*_2_ of CV_3 decreases to 167.6°C, indicating that the critical temperature required for the cell to evolve from early heat release to intense exothermic behavior has already dropped noticeably. For the cell CV_4, *T*_2_ decreases even further to 102.0°C, indicating that the cell enters an uncontrollable thermal instability stage at a temperature far below that of the baseline state. These results show that in commercial 12 Ah cells, *T*_2_ reflects the substantive influence of *D*_Li_ on the thermal runaway triggering threshold more effectively than *T*_1_. This observation is consistent with previous studies showing that Li‐plating intensifies side reactions between metallic Li or lithiated graphite and the electrolyte, accelerates interfacial destabilization, and thereby drives the cell into a rapid heat release stage at a lower temperature [33, 48]. In addition to the characteristic temperatures, the evolution time from the appearance of *T*_1_ to the triggering of *T*_2_ also shows a clear regularity. For CV_2, CV_3, and CV_4, the time intervals from the onset of *T*_1_ to thermal runaway triggering are approximately 1566.7, 1032.8, and 356.9 min, respectively, and decrease continuously with increasing *D*_Li_. This indicates that although cells with different *D*_Li_ all enter the early self‐heating stage at around 72°C, a higher *D*_Li_ leads to a much shorter buffer time before thermal runaway is triggered. As a result, thermal runaway evolves more rapidly, and the thermal safety margin becomes smaller. In particular, for CV_4, not only is *T*_2_ markedly advanced, but the time interval from *T*_1_ to *T*_2_ is compressed to about one‐third of that of CV_3, indicating that severe Li‐plating greatly weakens the tolerance of the cell to thermal perturbation and substantially increases the risk of thermal runaway.

**FIGURE 10:**
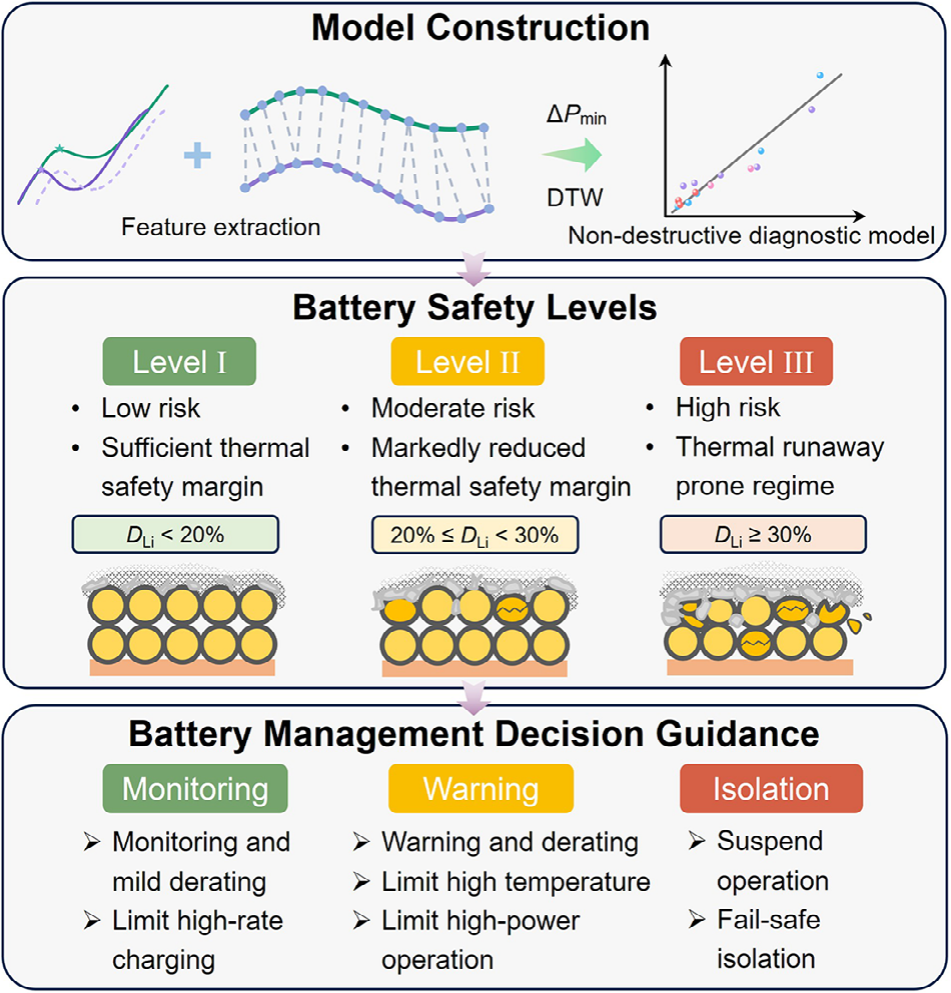
*D*_Li_‐based thermal safety grading and battery management framework.

These results demonstrate that the three thermal safety levels identified in this work remain applicable to commercial 12 Ah LFP pouch cells. Together with the results obtained from the low N/P cells, the consistent relationship between *D*_Li_ and thermal response across different cell scales and Li‐plating induction pathways indicates good cross‐scale transferability of the *D*_Li_‐based safety evaluation framework. Based on the *D*_Li_ values obtained from calibration pressure signals, the battery state can be classified into three safety levels, and corresponding management guidance is proposed, as shown in Figure 10.

Level I (*D*_Li_ < 20%): In this regime, the influence of Li‐plating and its accompanying side reactions on thermal behavior remains limited, and no pronounced decrease in the characteristic thermal temperature is observed. The cell retains a relatively sufficient thermal safety margin. Therefore, monitoring is recommended as the primary management strategy, together with mild derating when necessary. Measures such as limiting high‐rate charging, reducing residence time at high SOC, and optimizing the charge cutoff strategy can be applied to prevent further Li‐plating accumulation. The management objective at this level is to avoid crossing the 20% warning boundary and prevent the cell from entering the subsequent regime of rapidly increasing thermal risk.

Level II (20% ≤ *D*_Li_ < 30%): This regime corresponds to the stage in which *T*_f_ decreases at an accelerated rate with increasing *D*_Li_, indicating that the thermal safety margin becomes more sensitive to Li‐plating accumulation and that the risk of thermal runaway rises markedly. At this level, warning and derating strategies should be applied. Possible measures include limiting high power operation, reducing load under high temperature conditions, and conducting repeated diagnosis under the same calibration condition to reduce the influence of measurement noise and operational variation. The management objective at this level is to suppress further Li‐plating accumulation and prevent crossing the 30% critical threshold.

Level III (*D*_Li_ ≥ 30%): In this regime, *T*_f_ has entered a distinctly low temperature range, the thermal safety intervention window becomes substantially narrower, and unfavorable thermal responses are more likely to occur under external perturbations. Operation should be suspended, or fail‐safe isolation strategies should be triggered to reduce the probability of thermal runaway accidents. At this level, risk isolation should be treated as the primary management principle, and continued use should be avoided, especially under high temperature or high‐power conditions.

The proposed grading strategy is intended for cells in which degradation is dominated by Li‐plating and is used to reveal the quantitative relationship between *D*_Li_ and thermal behavior. In the commercial cell validation, *D*_Li_ is evaluated from pressure signals collected during the inserted C/10 calibration cycles after 4C cycling. Therefore, the framework focuses on the diagnosis of accumulated Li‐plating risk and the corresponding thermal safety grade based on calibration response, rather than on instantaneous warning during the high rate charging process. For broader application, further work can refine the relevant thresholds for systems involving multiple degradation sources by introducing additional diagnostic features and assessing cell‐to‐cell variability through replicated validation using commercial cells.

## 3 Conclusion

This work establishes an integrated framework that connects the quantitative diagnosis of Li‐plating degree with thermal safety grading for LIBs. Low N/P cells are constructed to induce controllable Li‐plating states, and *D*_Li_ is proposed as a quantitative descriptor of Li‐plating degree. Post‐mortem characterization shows that increasing *D*_Li_ is accompanied by the progressive accumulation of plated Li and reaction products, together with intensified interfacial and structural degradation. These observations support the use of *D*_Li_ to reflect Li‐plating accumulation and the associated degradation state. A non‐destructive diagnostic model is subsequently developed using DTW and normalized Δ*P*_min_ extracted from pressure signals collected during calibration cycles, enabling quantitative estimation of *D*_Li_.

ARC analysis further reveals the relationship between *D*_Li_ and battery thermal safety. Li‐plating accumulation significantly weakens the thermal safety margin of the cell, mainly manifested by the continuous decrease in the characteristic temperature *T*_f_ with increasing *D*_Li_. The segmented decrease in *T*_f_ provides the basis for thermal safety grading. *D*_Li_ = 20% corresponds to the transition from a gradual to an accelerated decrease in *T*_f_, and *D*_Li_ = 30% marks the transition to a distinctly lower *T*_f_ range with markedly increased thermal risk. Accordingly, these values are selected as the boundaries separating three thermal safety levels. Cross‐scale validation further demonstrates that the proposed *D*_Li_‐based safety evaluation remains applicable to commercial 12 Ah LFP pouch cells. In these cells, *T*_1_ is highly sensitive to the onset of early self‐heating induced by Li‐plating and can be used to identify whether the cell has entered a thermal degradation state governed primarily by Li‐plating. *T*_2_ and the evolution time from *T*_1_ to *T*_2_ exhibit stronger discrimination of *D*_Li_ and can more effectively characterize the progressive increase in thermal runaway risk.

Overall, this work integrates Li‐plating state construction, quantitative diagnosis, and thermal safety grading within a systematic research framework. It advances Li‐plating assessment from occurrence identification to degree quantification and corresponding thermal safety grading, providing methodological support for Li‐plating risk assessment and thermal safety management in LIBs.

## Author Contributions

**Wuliyasu He**: visualization, validation, formal analysis. **Jiangong Zhu**: writing – review and editing, funding acquisition, investigation, supervision. **Xuezhe Wei**: supervision, conceptualization. **Yang Wang**: visualization, validation, formal analysis. **Wenyuan Weng**: visualization, validation, investigation. **Wenjun Fan**: visualization, validation, formal analysis. **Bin Shen**: visualization, validation, formal analysis. **Huapeng Lu**: visualization, validation, formal analysis. **Chunjing Lin**: visualization, validation, formal analysis. **Haifeng Dai**: supervision, conceptualization. **Haonan Liu**: writing – original draft, investigation, data curation, formal analysis.

## Conflicts of Interest

The authors declare no conflicts of interest.

## Supporting information




**Supporting File**: advs77129‐sup‐0001‐SuppMat.docx.

## Data Availability

The data that support the findings of this study are available from the corresponding author upon reasonable request.
