# Supplementary material for: Lithium‐Ion Battery Safety Evaluation by Quantifying Lithium Plating Degree Based on Pressure Signals
Source: Adv Sci (Weinh). 2026 Aug 11:e77129. Online ahead of print. doi: 10.1002/advs.77129 (PMC13460271; doi:10.1002/advs.77129)
Supplement: Supplementary file 1 [file ADVS-9999-e77129-s001.docx]

Supplementary Information

**Lithium-ion battery safety evaluation by quantifying lithium plating degree based on pressure signals**

Haonan Liu, Jiangong Zhu^*^, Wenyuan Weng, Bin Shen, Wenjun Fan, Yang Wang, Huapeng Lu, Wuliyasu He, Chunjing Lin, Xuezhe Wei, Haifeng Dai

**Experiments**

**Cell fabrication.** The cells used in this study were self-made pouch cells, with LiFePO_4_ (LFP) as the cathode and graphite as the anode. The N/P ratio of the cells was controlled at 0.65 by adjusting the areal loading of the anode, and the detailed electrochemical parameters were shown in Table S1. The pouch cells were fabricated in a Z-folding stacking configuration with 14 graphite anode sheets and 13 LFP cathode sheets, separated by the polyethylene (PE) separator coated with alumina (Al_2_O_3_). After drying for 12 h, the cells were assembled in an argon-filled glove box (MBRAUN, H_2_O < 0.1 ppm and O_2_ < 0.1 ppm) and injected with 5 g of electrolyte (KLD-LFP01) to complete vacuum sealing. The cells were left to rest for 12 h to allow sufficient electrolyte wetting, followed by two formation cycles at C/20 to reach a stable initial state.

**Electrochemical test.** The N/P = 0.65 cells were placed in devices for operando pressure measurements (Figure S1). The cells were fixed between two aluminum alloy plates, allowing any variation in the thickness of the electrode stack to be directly reflected in the pressure exerted on the plates. An additional aluminum alloy plate was inserted between the pressure sensor and the cell. Two soft foam layers with a thickness of 3 mm were used as buffer pads, one placed between the back side of the cell and the aluminum alloy plate, and the other between the front side of the cell and the additional aluminum alloy plate. The soft foam layers were introduced to improve contact conformity between the pouch cell and the pressure plates, owing to the inherent flexibility and local thickness variations of pouch cells. This configuration reduces local stress concentration and helps transfer cell thickness changes to the pressure plates in a more uniform manner. This design ensured uniform loading on the cell while preventing damage to the electrode sheets. Each device was secured by four bolts, and the initial pressure applied to the cell was regulated by adjusting the bolts. Before cycling, an initial preload of 70 kPa (200 N) was applied to the cell at 0% state of charge (SOC) and maintained for 24 h to allow stress relaxation. During this period, the bolts were repeatedly adjusted until the cell pressure stabilized at the initial preload level. This preload stabilization step was used to reduce the influence of initial foam relaxation before electrochemical testing. All cells were tested with the same foam thickness, plate configuration, preload value, and resting procedure, so that any remaining buffer-layer effect was kept consistent among different samples. In addition, the pressure analysis in this work was based on relative pressure response ΔP, IC/FC comparison, and curve alignment, rather than direct comparison of absolute pressure values. These treatments reduce the influence of baseline drift and slow stress relaxation on the extracted pressure features.

To ensure sufficient utilization of the cathode capacity while avoiding entry of the anode into the Li-plating regime, the voltage window was set between 2.5 V and 3.38 V [1]. Before formal testing, the N/P = 0.65 cells were subjected to two initial cycles at C/5. The average discharge capacity of these cycles was defined as the rated capacity (Q), which served as the reference for the depth of overcharge. By adjusting the charge cutoff capacity, the terminal charge capacity was sequentially set to 100%Q, 115%Q, 125%Q, 135%Q, and 150%Q to induce Li-plating of different degrees under controlled conditions, thereby yielding one standard condition and four overcharge conditions. For the overcharge conditions, the upper cutoff voltage was limited to 5.0 V, which was the maximum voltage allowed by the testing system. For simplicity, these five cycling conditions were denoted as Q, 1.15Q, 1.25Q, 1.35Q, and 1.50Q, respectively. All five conditions were cycled at 1C, while calibration was carried out at C/10. This study focused on the degradation and safety risks caused by Li-plating. Therefore, the cycling tests were limited to a maximum of 50 cycles or terminated upon cell failure.

**Post-mortem characterization.** All cells were disassembled in the argon-filled glovebox for post-mortem observation of Li-plating. The harvested anodes, cathodes, and separators were then immersed in dimethyl carbonate (DMC) solution to remove residual electrolyte and dried in the glovebox before characterization. Surface morphology was characterized by scanning electron microscopy (SEM, Regulus 8100), and cross-sectional morphology was analyzed using a cross-section polisher (CP, IM4000 II). Chemical composition was characterized by X-ray photoelectron spectroscopy (XPS, PHI 5000 VersaProbe III), with measurements performed on the sample surface and at a depth of approximately 30 nm. The heat flow response of the electrode samples during heating was measured by differential scanning calorimetry (DSC, Mettler Toledo TGA/DSC 3+). All samples were transferred using a vacuum transfer chamber.

**Thermal safety test.** Five more N/P = 0.65 cells were subjected to the same formation and two initial cycles, and then charged to 100%Q, 110%Q, 120%Q, 130%Q, and 140%Q, respectively. In addition, several commercial 12 Ah LFP pouch cells with a conventional N/P ratio were selected, and samples with different Li-plating degrees were constructed through different cycling conditions, as described in Section 3.5. After reaching the target states, both types of cells were rested for 2 h and then tested using an extended-volume accelerating rate calorimeter (EV+ARC) produced by Thermal Hazard Technology (THT) and operated in heat-wait-seek (HWS) mode. For the EV+ARC tests, the start temperature was set to 40 °C, the heat step temperature was 5 °C, and the temperature rate sensitivity was 0.02 °C/min. Considering the differences in thermal response between cells of different scales, the waiting time was set to 30 min for the N/P = 0.65 cells and 45 min for the 12 Ah commercial cells.

Figure S1. Pressure fixture.

Figure S2. Anode charging voltage 0 V vs. Li/Li^+^ at overcharge depths of 1-1.50Q.

Figure S3. Comparison of the capacity fade curves.

Figure S4. Morphological evolution of graphite anodes. (a, b) Optical images, (c, d) surface SEM, and (e, f) cross-sectional SEM.

Figure S5. Morphological evolution of cathodes and separator. (a, d) Q, (b, e) 1.25Q, and (c, f) 1.50Q.

Figure S6. XPS spectra of graphite anodes at 0nm and 30 nm depth. (a-f) 1.15Q, (g-l) 1.35Q.

Figure S7. The variations of unit lattice volume with the stoichiometric coefficient x [2]. (a) Graphite, (b) LiFePO_4_.

Figure S8. Pressure curves under IC and FC conditions.

**Note S1 Detailed calculation procedure of DTW.**

The dynamic time warping (DTW) algorithm is widely used to calculate the optimal warping path between two time series [3]. Its core idea is to allow nonlinear stretching and compression along the time axis while preserving path monotonicity and continuity, so as to identify the matching path that minimizes the overall distance metric. As illustrated in Figure S9, DTW is employed here to quantify the distance between the relative expansion pressure curve of the calibration cycle, Δ*P*_FC_ (*y*_FC,1_, *y*_FC,2_, *y*_FC,3_… *y*_FC,_*_n_*), and the initial baseline curve, Δ*P*_IC_ (*y*_IC,1_, *y*_IC,2_, *y*_IC,3_… *y*_IC,_*_m_*), where *m* and *n* denote the lengths of the two pressure sequences, respectively. Before calculating the DTW distance, the two pressure sequences are separately subjected to Z-score standardization. Specifically, each sequence is standardized by subtracting its own mean and dividing by its own standard deviation. This treatment reduces differences in pressure offset and amplitude scale while retaining the curve profile along the normalized capacity axis. For simplicity, the standardized sequence values are still denoted as *y*_FC,_ *_i_* and *y*_IC,_ *_j_* in the following formulation. By constructing a matrix *S* and applying dynamic programming, DTW searches for the minimum accumulated distance among all admissible paths and thus obtains the DTW distance as a measure of the similarity between the two curves. Specifically, the procedure consists of three steps: first, the start and end points of the alignment path are initialized and a two-dimensional matrix *S* is defined; second, the value of each element in *S* is recursively calculated, and the minimum cumulative distance is selected according to Equation (1); finally, the optimal path is traced back to obtain the minimum distance metric.

where$\text{d}_{i,j}$ denotes the distance between *y*_FC,_ *_i_* and *y*_IC,_ *_j_*. This dynamic programming procedure guarantees path monotonicity and continuity while searching for the global minimum cumulative distance.

Figure S9. Schematic of the DTW algorithm.

Figure S10. DSC results. (a) The anodes of Q and 1.4Q without electrolyte. (b) The PE separator coated with Al_2_O_3_.

where *n* denotes the number of sampling points, $\Delta P_{i}^{''}$ represents the value of the i-th sampling point.

where *n* is the total number of calibration cycles for all samples, $y_{i}$ denotes the measured *D*_Li_ of the i-th calibration cycle, $\hat{y}_{i}$ denotes the predicted *D*_Li_ of the i-th calibration cycle, and $\bar{y}$ is the average of the measured *D*_Li_ over all calibration cycles.

| **Table S1**  Battery basic parameters. |  |
| --- | --- |
| Parameter | N/P = 0.65 |
| Cathode specific capacity (mAh/g) | 145 |
| Cathode areal loading (mg/cm^2^) | 17 |
| Cathode active material content (%) | 96 |
| Anode specific capacity (mAh/g) | 345 |
| Anode areal loading (mg/cm^2^) | 4.6 |
| Anode active material content (%) | 96.4 |

| **Table S2**  Comparison of two-feature combinations for *D*_Li_ diagnosis. | | | |
| --- | --- | --- | --- |
| Feature combination | R^2^ | RMSE (%) | MAE (%) |
| DTW + Δ*P*_min_ | 0.90 | 3.02 | 2.49 |
| DTW + Δ*P*_mean_ | 0.84 | 3.44 | 2.71 |
| DTW + Δ*P*_var_ | 0.83 | 3.75 | 2.87 |

References

[1] Liu H, Zhu J, Shen B, Weng W, Xu W, Wang Y, et al. Investigation of lithium plating mechanism based on N/P ratio controlled lithium-ion batteries. Applied Energy. 2026;408:127427 <https://doi.org/10.1016/j.apenergy.2026.127427>.

[2] Ding S, Wang L, Dai H, He X. Prognosticating nonlinear degradation in lithium-ion batteries: operando pressure as an early indicator preceding other signals of capacity fade and safety risks. Energy Storage Materials. 2025;75:103998 <https://doi.org/10.1016/j.ensm.2024.103998>.

[3] Shuai C, Sun Y, Zhang X, Yang F, Ouyang X, Chen Z. Intelligent Diagnosis of Abnormal Charging for Electric Bicycles Based on Improved Dynamic Time Warping. IEEE Transactions on Industrial Electronics. 2023;70:7280-9 <https://doi.org/10.1109/TIE.2022.3206702>.
